# Clinical Significance of Assessment of Thrombospondin and Placenta Growth Factor Levels in Patients with Sickle Cell Anemia: Two Centers Egyptian Studies

**DOI:** 10.4084/MJHID.2014.044

**Published:** 2014-07-01

**Authors:** Adel A Hagag, Ghada Elmashad, Aml Ezzat Abd El-Lateef

**Affiliations:** 1 Pediatrics Department, Faculty of Medicine, Tanta University, Egypt; 2 Clinical Pathology Department, Faculty of Medicine, Tanta University, Egypt; 3 Pediatric Department, Faculty of Medicine, Elmenofia University, Egypt

## Abstract

**Background:**

Sickle cell disease has a worldwide distribution. Vaso-occlusive crisis (VOC) is one of the most important clinical features of the disease. Thrombospondin (TSP1) and Placenta growth factor (PlGF) have been reported to be involved in sickle cell diseases (SCD).

**Objective:**

The aim of this study was to assess the clinical significance of Thrombospondin and Placenta growth factor profiles in patients with sickle cell disease.

**Patients and methods:**

This study was carried out in sixty patients with sickle cell anemia who were attendants to Hematology units, Pediatric Departments, Tanta and Elmenofia University Hospitals in the period between December 2011 and May 2014 including thirty patients during vaso-occlusive crisis and thirty patients out of crisis. Also this study included twenty healthy children of matched age and sex as a control group. Serum TSP1 and PlGF levels were analyzed by ELISA.

**Results:**

In SCA patients with crisis the mean serum Thrombospondin level was 902.5±280.89 ng/mL; in SCA patients out of crisis the mean serum Thrombospondin level was 462.5 ± 190.2 ng/mL and in controls the mean value was 236.66±58.29 ng/mL. In SCA patients with crisis the mean serum Placenta growth factor level was 19.97±1.28 pg/ml; in SCA patients out of crisis the mean serum Placenta growth factor level was 13.12 ± 1.82 pg/ml and in controls the mean value was 9.89 ± 1.20 pg/ml. All paired comparisons for Thrombospondin and Placenta growth factor reached statistical significance (P< 0.001). There was significant positive correlation between serum Thrombospondin and Placenta growth factor levels in sickle cell anemia patients during crisis (r=0.848, p=<0.001).

**Conclusions:**

This is the first study to show TSP1and PlGF concentration changes in patients with SCD in a large cohort study from Middle East, and to show correlation between both markers; therefore TSP1and PlGF may be useful VOC markers in SCD patients.

**Recommendation:**

To further assess TSP1 and PlGF as a marker of VOC in patients with SCD, further studies should be conducted to determine the exact point before VOC, when serum TSP1 and PIGF levels begin to increase. This requires monitoring of the TSP1 and PIGF levels in sickle cell patients out of crisis, showing how rapidly these levels increase just before VOC development.

## Introduction

Sickle cell disease (SCD) is hereditary hemoglobinopathy characterized by abnormal hemoglobin production, hemolytic anemia, and intermittent occlusion of small vessels, leading to acute and chronic tissue ischemia, chronic organ damage, and organ dysfunction.^1^ Sickle hemoglobin (Hb S) is common and clinically significant hemoglobin structural variant.^2^

Hb S is caused by β-globin gene mutation in which the 17^th^ nucleotide is changed from thymine to adenine and the 6^th^ amino acid in the β-globin chain becomes valine instead of glutamic acid; this mutation produces a hydrophobic motif in the deoxygenated Hb S tetramer that results in binding between β_1_ and β_2_ chains of two hemoglobin molecules. This crystallization produces a polymer nucleus, which grows and fills the erythrocyte, disrupting its architecture and flexibility and promoting cellular dehydration.^3^ Damage to the erythrocyte cell membrane occurs as it passes through the microcirculation, shortening its life span and causing chronic hemolytic anemia.^1^ Also Hb S polymerizes when sickle RBCs are exposed to hypoxic conditions in the microcirculation, leading to increased cellular adhesiveness, nitric oxide depletion and vaso-occlusion.^4^ Most patients will have severe pain due to occlusion of blood flow to bones, muscles, arms, legs, back, abdomen, and chest.^5^

Cytokines and adhesion molecules play an important role in the pathophysiology of vaso-occlusion in SCD.^6^ Placenta growth factor (PlGF) is released by immature erythrocytes and is elevated in SCD and may play a role in the pathophysiology of sickle cell disease-associated pulmonary hypertension by inducing the release of vasoconstrictor substance called endothelin-1.^7^

Platelets are activated in SCD particularly during vaso-occlusive episodes (VOE).^8^ Increased platelet activation likely plays a catalytic role in vaso-occlusion and vasculopathy in SCD 9,10 by increasing the adhesion of sickle RBCs to the endothelium^11^ via secretion of fibrinogen, von Willebrand Factor^12^ and Thrombospondin-1 (TSP1)^13^ and promoting further intimal damage.^14^

TSP1 is multifunctional glycoprotein containing domains for adhesive proteins, enzymes, cell receptors that is abundantly present in platelet α-granules, and is a key player in vascular biology.^15^ TSP1 is the major secretory product of activated platelets, which is increased in VOE.^8^ TSP1, via its cognate receptor CD47, modulates vascular responses to hypoxia, regulates vaso-constriction, inhibits angiogenesis, and promotes adhesion of sickle RBCs to the endothelium.^16^ Moreover, TSP1 inhibits NO signaling pathway through binding to the receptors CD36 and CD47 expressed on endothelial cells and platelets ^(17, 18)^ thus; TSP1 represents a plasma biomarker of disease severity in SCD.^8^

## Aim of this Study

The aim of this study was to assess the clinical significance of Thrombospondin and Placenta growth factor profiles in patients with sickle cell disease during crisis and in steady state.

## Patients and Methods

This study was done after approval from ethical committee of research center in Tanta and Elmenofia University Hospitals and informed written parental consent from every case that participates in this research and was carried out on 60 cases with sickle cell disease (HbSS) who were admitted or under follow up at Hematology unit, Pediatric department, Tanta and Elmenofia University Hospitals in the period between December 2011 and May 2014, including thirty patients with sickle cell anemia during vaso-occlusive crisis (18 males and 12 females) and thirty patients in steady state out of crisis (15 males and 15 females). Also this study included twenty healthy children of matched age and sex as a control group. To ensure that the patients is not in crisis samples were obtained from patients who had no acute sickle events, fever, or infections 3 weeks before or 3 weeks after the blood sample and were not transfused within the last 90 days.^19^ Vaso-oclusive crisis is acute painful condition at any site of the patient’s body due to occlusion of blood flow to bones, bone marrow, muscles, organs, arms, legs, back, abdomen, or chest ^5.^

### For all patients the following were done

- Complete history taking
- Thorough clinical examination with especial account on pallor, jaundice, leg ulcers, hepatomegaly and splenomegaly.
- Laboratory investigations including:
  - *Complete blood count.* One ml of venous blood were collected using sterile needles through gentle venipuncture after sterilization of site of puncture by alcohol, and collected samples were delivered on 20 uL EDTA solution for complete blood count including reticulocytic count and differential count which was done on leishman stained peripheral blood smear with evaluation using ERMA PCE-210 N cell –counter.^20^
  - *Serum thrombospondin levels.* Two ml of venous blood samples from patients and controls were collected in citrated tubes and immediately transferred to laboratory at 4°C. The tubes were inverted 8–10 times and then subjected to double centrifugation at 1500g at 4°C to obtain platelet poor plasma (PPP). The supernatant was aliquoted into cryotubes and stored at −80°C until the day of testing by ELISA. PPP were thawed and assessed for levels of TSP1 by ELISA in duplicate (R&D Systems, Minneapolis, MN).^8^
  - *Serum placenta growth factor levels.* Two ml of Heparinized venous blood samples was obtained from patients with SCD and healthy controls. The blood samples were centrifuged at 0°C – 4°C and 1000*g* for 15 minutes and plasma was separated within 2 hours of sample collection and stored at −80°C until it was assayed. PlGF concentration was determined on cell-free heparinized plasma using ELISA.^19^

### Statistical analysis

Data were collected and analyzed using SPSS for windows (version 12). All Data were expressed as in terms of mean values ± SD. Comparisons of parameters among groups were made using the paired t test. Two-group comparisons were performed non-parametrically using the Mann-Whitney U test. All statistical tests were two tailed, and *P* < 0.05 was considered statistically significant.

## Results

There were no statistically significant differences between sickle cell anemia patients with and without vaso-occlusive crisis as regards age, sex, pallor, jaundice, leg ulcers, hepatomegaly and splenomegaly (Table 1).

There were statistically significant differences between patients with or without VOC and control group as regards platelets; RBCs and WBCs but there were no statistically significant differences between patients with and without VOC (Table 2).

Mean serum Thrombospondin levels were significantly higher in sickle cell anemia patients with crisis than those out of crisis and were significantly higher in sickle cell anemia patients with or without crisis than control group (Table 3).

Mean serum Placenta growth factor levels were significantly higher in sickle cell anemia patients with crisis than sickle cell anemia patients out of crisis and were significantly higher in SCA patients with or without crisis than controls (Table 3).

**Figure 1:**
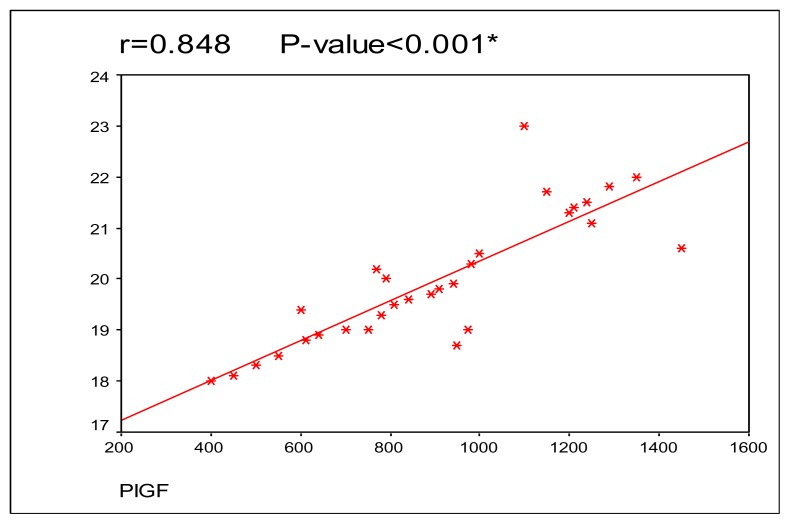
Correlation between thrombospondin and placenta growth factor levels in patients with sickle cell anemia with vaso-occlusive crisis.

Significant positive correlation was found between serum Thrombospondin and Placenta growth factor levels in sickle cell anemia patients during crisis **(**Figure 1).

## Discussion

Sickle cell disease is one of the most important single gene disorders of human beings^21^ that affects 1/400 individuals of African descent, as well as people of Arab, Indian and Hispanic descents.^22^ VOC has a complex nature, involving interactions between sickle red blood cells, endothelium, and leucocytes. Endothelial damage due to recurrent adhesion of sickle RBCs may disrupt endothelial function, leading to altered cytokine release. Altered balance of proinflammatory and anti-inflammatory cytokines plays an important role in a painful crisis in SCD patients.^23^ Placenta growth factor is angiogenic growth factor released by immature erythrocytes and is elevated in SCD.^24^,^25^ Thrombospondin-1 is the major secretory product of activated platelets and is a key player in vascular biology that is increased in VOE.^8^,^15^

In this study Thrombospondin and Placenta growth factor were measured by a commercially available ELISA kits in 60 sickle cell disease patients including 30 cases in steady state and 30 cases in a painful crisis compared with 20 normal controls.

In the present study mean, serum Thrombospondin levels were significantly higher in SCA patients with crisis than patients out of the crisis and were significantly higher in SCA patients with or without crisis than controls. This datum was in agreement with Novelli et al 2013^26^ who found the same results and Novelli et al 2012^8^ who tested 27 patients in steady state and 14 patients with VOE, as well as 17 healthy controls and found the same results with a positive correlation between TSP-1 levels and vaso-occlusive complications and history of acute chest syndrome^8^ and explained this by increased platelet activation and degranulation, that can lead to increased plasma levels of TSP1 in patients with sickle cell anemia with or without crisis, in accordance with a prior study that showed increased platelet activation in VOE.^10^

In the current study placenta, growth factor levels were significantly higher in SCA patients with crisis than patients out of crisis and were significantly higher in SCA patients with or without crisis than controls. This datum was in agreement with Bottomley et al 2000,^27^ Natalya et al 2003,^19^ Nitin et al 2009^28^ and Nitin et al 2010^29^ who found significant positive correlation between PlGF concentrations and incidence of VOC and they concluded that PlGF could contribute to vascular occlusion and might modulate clinical severity, since PlGF causes a significant increase in proinflammatory cytochemokines mRNA in monocytes.^19^ These proinflammatory molecules contributed to the activation of leukocytes and endothelial cells, a phenomenon observed in SCD at steady state,^30^ and may be responsible for the increased incidence of vascular occlusions in SCD subjects. The leukocytes adhesion to endothelium is a primary event in initiating vascular occlusion and secondarily causes RBCs to adhere to leukocytes or to endothelium.^31^ Brittain et al 2010^32^ found significantly elevated PlGF in SCD compared with healthy controls but did not observe any association of PlGF with the frequency of acute pain episodes or history of acute chest syndrome.

In this work, there were significant positive correlations between serum TSP-1 and PlGF levels in patients with sickle cell anemia during vaso-occlusive crisis. This study is, to our knowledge, the first to correlate these two parameters. The significant positive correlation between serum TSP-1 and PlGF levels in this study could be explained by hypoxia, which was shown to be a strong stimulus for angiogenesis in numerous disorders including sickle cell anemia.^33^ Hypoxia inducible transcription factors induce the expression of several angiogenic factors including VEGF, nitric oxide synthase, PlGF and TSP-1.^33^ Both of TSP-1 and PlGF increase together during hypoxia in sickle cell anemia especially in vaso-occlusive crisis.^33^ Also, Placenta growth factor is released by immature erythrocytes and is elevated in SCD due to hyperactive bone marrow. Concomitantly Thrombospondin is released from activated platelets in case of SCD particularly during vaso-occlusive episodes.^7^,^8^

On the basis of our results, we concluded that the increased TSP1 and PIGF levels could be considered as a marker of VOC in SCD. Further studies should be performed to determine the exact timing of TSP1 and PIGF levels increase, in relation to the episodes of VOC. The monitoring of TSP1 and PIGF levels in patients with sickle cell out of the crisis appears necessary to this scope.

## Figures and Tables

**Table 1:** Clinical data of studied patients with sickle cell disease.

Patients data	Sickle cell anemia (HbSS) with VOC (n=30)	Sickle cell anemia (HbSS) out of crisis (n=30)	X2	P
**Sex**				
Males	18 (60%)	15 (50%)	2.99	0.39
Females	12 (40%)	15 (50%)	1.48	0.25
**Age**				
Mean age (range) in years	8.86 ± 2.74 (7–11)	10.30 ± 4.05 (8–12)	1.47	0.15
**Clinical manifestations**				
Pallor	24 (80%)	21 (70%)	0.14	0.70
Jaundice	21 (70%)	22 (73.3%)	0.26	0.22
Hepatomegaly	23 (76.6%)	24 (80%)	0.23	0.88
Splenomegaly	20 (66.6%)	19 (63.3%)	0.11	0.73
Leg ulcers	2 (6.66%)	1 (3.33%)	0.97	0.55

**Table 2:** Comparison between sickle cell anemia patients with or without crisis and control group regarding complete blood picture.

Parameters	Sickle cell anemia with VOC (n=30)	Sickle cell anemia out of crisis (n=30)	Control (n=20)
**WBCS** ®			
Mean (range)	11.8 ± 3.91(6.7–18.5)	10.74± 3.25 (7–18)	6.72±2.51(4–12)
T value	1.12‡	3.44^0^	3.22*
P value	0.96‡	0.006^0^	0.008*
**Platelets**®			
Mean (range)	359.6 ± 32.45 (160–650)	357.65 ± 35.71(170–680)	292± 18.75(150–420)
T value	0.75‡	3.22^0^	4.52*
P value	0.11‡	0.01^0^	0.03*
**RBCS** ©			
Mean (range)	2.3± 0.3 (2.3–2–7)	2.57±0. 2(2.3–3)	3.7–4.3))3.72 ± 0.22
T value	0.96‡	2.88^0^	2.66*
P value	0.63‡	0.028^0^	0.04*

® WBCs and platelets in thousands/mm^3^,

© RBCS in million/mm^3^.

* SCD with crisis versus control,

0 SCD out of crisis versus control,

‡ SCD with crisis versus out of crisis.

**Table 3:** Comparison between serum levels of Thrombospondin and PIGF in Sickle cell anemia with or without crisis and control group.

Parameters	Sickle cell anemia with VOC (n=30)	Sickle cell anemia out of crisis (n=30)	Control (n=20)
**Thrombospondin (ng/mL)**			
Mean (range)	902.5±280.89(400–1450)	462.50± 190.20 (190–900)	236.66±58.29(130–320)
T value	8.83‡	5.93^0^	13.24*
P value	<0.001‡	<0.001^0^	<0.001*
**PIGF (pg/ml)**			
Mean (range)	19.97±1.28 (15–23)	13.12± 1.82 (11–18)	9.89 ± 1.20 (8–13)
T value	34.37‡	16.65^0^	8.21*
P value	<0.001‡	<0.001^0^	<0.001*

* SCD with crisis versus control,

0 SCD out of crisis versus control,

‡ SCD with crisis versus out of crisis.

## References

[1] Smiley D, Dagogo-Jack S, Umpierrez G (2008). Therapy insight: metabolic and endocrine disorders in sickle cell disease. Nat Clin Pract Endocrinol Metab.

[2] Piel FB, Patil AP, Howes RE, Nyangiri OA, Getting PW, Dewi M, Temperley WH, Williams TN, Weatherall DJ, Hay SI (2013). Global epidemiology of sickle haemoglobin in neonates: a contemporary geostatistical model-based map and population estimates. Lancet.

[3] Rees DC, Williams TN, Gladwin MT (2010). Sickle cell disease. Lancet.

[4] Stuart MJ, Nagel RL (2004). Sickle-cell disease. Lancet.

[5] Roseff SD (2009). Sickle cell disease: a review. Immuno-hematology.

[6] Schnog JB1, Rojer RA, Mac Gillavry MR, Ten Cate H, Brandjes DP, Duits AJ (2003). Steady-state sVCAM-1 serum levels in adults with sickle cell disease. Annals of Hematology.

[7] Britain JE, Hulkower B, Jones SK, Strayhorn D, De Castro L, Telen MJ, Orringer EP, Hinderliter A, Ataga KI (2010). Placenta growth factor in sickle cell disease: association with hemolysis and inflammation. Blood.

[8] Novelli EM, Kato GJ, Ragni MV, Zhang Y, Hildesheim ME, Nouraie M, Barge S, Meyer MP, Hassett AC, Gordeuk VR, Gladwin MT, Isenberg JS (2012). Plasma thrombospondin-1 is increased during acute sickle cell vaso-occlusive events and associated with acute chest syndrome, hydroxyurea therapy, and lower hemolytic rates. American Journal of Hematology.

[9] Hu W1, Jin R, Zhang J, You T, Peng Z, Ge X, Bronson RT, Halperin JA, Loscalzo J, Qin X (2010). The critical roles of platelet activation and reduced NO bioavailability in fatal pulmonary arterial hypertension in a murine hemolysis model. Blood.

[10] Villagra J1, Shiva S, Hunter LA, Machado RF, Gladwin MT, Kato GJ (2007). Platelet activation in patients with sickle disease, hemolysis- associated pulmonary hypertension, and nitric oxide scavenging by cell-free hemoglobin. Blood.

[11] Antonucci R, Walker R, Herion J, Orringer E (1990). Enhancement of sickle erythrocyte adherence to endothelium by autologous platelets. Am J Hematol.

[12] Blann AD1, Marwah S, Serjeant G, Bareford D, Wright J (2003). Platelet activation and endothelial cell dysfunction in sickle cell disease is unrelated to reduced antioxidant capacity. Blood Coagul Fibrinolysis.

[13] Brittain HA1, Eckman JR, Swerlick RA, Howard RJ, Wick TM (1993). Thrombospondin from activated platelets promotes sickle erythrocyte adherence to human microvascular endothelium under physiologic flow: A potential role for platelet activation in sickle cell vaso-occlusion. Blood.

[14] Schermuly RT1, Dony E, Ghofrani HA, Pullamsetti S, Savai R, Roth M, Sydykov A, Lai YJ, Weissmann N, Seeger W, Grimminger F (2005). Reversal of experimental pulmonary hypertension by PDGF inhibition. J Clin Invest.

[15] Bonnefoy A, Moura R, Hoylaerts MF (2008). The evolving role of thrombospondin-1 in hemostasis and vascular biology. Cell Mol Life Sci.

[16] Roberts DD, Miller TW, Rogers NM, Yao M, Isenberg JS (2012). The matri-cellular protein thrombospondin-1 globally regulates cardiovascular function and responses to stress via CD47. Matrix Biol.

[17] Isenberg JS1, Ridnour LA, Perruccio EM, Espey MG, Wink DA, Roberts DD (2005). Thrombospondin-1 inhibits endothelial cell responses to nitric oxide in a cGMP-dependent manner. Proc Natl Acad Sci USA.

[18] Isenberg JS1, Ridnour LA, Dimitry J, Frazier WA, Wink DA, Roberts DD (2006). CD47 is necessary for inhibition of nitric oxide- stimulated vascular cell responses by thrombospondin-1. J Biol Chem.

[19] Perelman Natalya, Selvaraj Suresh K, Batra Sandeep, Luck Lori R, Erdreich-Epstein Anat, Coates Thomas D, Kalra Vijay K, Malik Punam (2003). Placenta growth factor activates monocytes and correlates with sickle cell disease severity. Blood.

[20] Monica C, Tito A, Lewis SM Strategy of blood safety in Africa region. District laboratory practice in tropical countries.

[21] Jastaniah W (2011). Epidemiology of sickle cell disease in Saudi Arabia. Ann Saudi Med.

[22] Alexya T, Sangkatumvongb S, Connesd P (2010). Sickle cell disease: Selected aspects of pathophysiology. Clin Hemorheol Microcirc.

[23] Pathare A1, Al Kindi S, Alnaqdy AA, Daar S, Knox-Macaulay H, Dennison D (2004). Cytokine profile of sickle cell disease in Oman. Am. J. Hematol.

[24] Tordjman R1, Delaire S, Plouët J, Ting S, Gaulard P, Fichelson S, Roméo PH, Lemarchandel V (2001). Erythroblasts are a source of angiogenic factors. Blood.

[25] Green CJ, Lichtlen P, Huynh NT, Yanovsky M, Laderoute KR, Schaffner W, Murphy BJ (2001). Placenta growth factor gene expression is induced by hypoxia in fibroblasts: a central role for metal transcription factor-1. Cancer Res.

[26] Novelli EM, Kato GJ, Hildesheim ME, Barge S, Meyer MP, Lozier J, Hassett AC, Ragni1 MV, Isenberg JS, Gladwin MT (2013). Thrombospondin-1 inhibits ADAMTS13 activity in sickle cell disease. Haematologica.

[27] Bottomley MJ1, Webb NJ, Watson CJ, Holt L, Bukhari M, Denton J, Freemont AJ, Brenchley PE (2000). PlGF induces vascular endothelial growth factor (VEGF) secretion from mononuclear cells and is co-expressed with VEGF in synovial fluid. Clin Exp Immunol.

[28] Patel Nitin, Gonsalves Caryn S, Yang Minyang, Malik Punam, Kalra Vijay K (2009). Placenta growth factor induces 5-lipoxygenase-activating protein to increase leukotriene formation in sickle cell disease. Blood.

[29] Patel N, Sundaram N, Yang M, Madigan C, Kalra VK, Malik P (2010). Placenta growth factor (PlGF), a novel inducer of plasminogen activator inhibitor-1 (PAI-1) in sickle cell disease (SCD). J Biol Chem.

[30] Wun T, Cordoba M, Rangaswami A, Cheung AW, Paglieroni T (2002). Activated monocytes and platelet-monocyte aggregates in patients with sickle cell disease. Clin Lab Haematol.

[31] Turhan A, Weiss LA, Mohandas N, Coller BS, Frenette PS (2002). Primary role for adherent leukocytes in sickle cell vascular occlusion: a new paradigm. Proc Natl Acad Sci U S A.

[32] Britain JE, Hulkower B, Jones SK, Strayhorn D, De Castro L, Telen MJ, Orringer EP, Hinderliter A, Ataga KI (2010). Placenta growth factor in sickle cell disease: association with hemolysis and inflammation. Blood.

[33] Gupta K, Zhang J (2005). Angiogenesis: a curse or cure?. Postgrad Med J.

